# Summer Diarrhœa of Children—Its Etiology and Rational Treatment*Read before Tri-State Medical Society of Georgia, Alabama and Tennessee, October 1891.

**Published:** 1891-12

**Authors:** E. F. Camp

**Affiliations:** Gadsden, Ala.


### SUMMER DIARRHŒA OF CHILDREN—ITS ETIOLOGY AND RATIONAL TREATMENT.*

BY E. F. CAMP, M. D., GADSDEN, ALA.

When I was requested by our worthy secretary to prepare a paper for the Tri-State Medical Association, it was some little time before I could get my consent to do so; and when this was accomplished, there arose another obstacle; What shall I write about? After this was decided there arose still another important question; What shall, or what can I say on the subject?

When we consume the time and ask the attention of such an intelligent representative body of medical talent as is here assembled, not only of the three great States composing the Tri-State Medical Association, but from various other States, we should have something of importance to say.

I cannot say whether or not I shall be able to say anything of importance, or advance an idea that is not familiar, to at least a majority of you. But I do claim the subject to be an important one. It is important, first, because every general

*Read before Tri-State Medical Society of Georgia, Alabama and Tennessee. October 1891.

practitioner has to battle with this deadly enemy, let him locate where he will, in the crowded city, in town, hamlet or country cross-roads, he has this monster to meet. Second, it is important because it claims for its victims a large per cent. of those who are most near and dear to us. Third, it is important because, until very recently, its true cause was not fully understood, and I cannot say that it is fully understood even now, notwithstanding the immense amount of modern light that has been thrown upon the subject. Fourth, it is important because there is no disease that is so prevalent which has been so irrationally treated in former years; and is still being so treated by some of the prominent members of the profession.

Before entering into the etiology or treatment of this disease, I may say that in the term Summer Diarrhoea is included the catarrhal conditions found in different portions of the gastro-intestinal tract and between different grades as entero-colstis, and cholera-infantum, considering them as varying only in degree and in intensity.

To treat any disease successfully, we should understand its whole nature, especially its etiology. We recognize three principal etiological factors in this disease, namely: Improper food, high atmospheric temperature and micro-organism. I have named them in the order of their importance. This is proven by statistics of different observers on the subject.

Of 1943 fatal cases observed by Siebert of New York, only sixty-one, or about three per cent, were breast fed exclusively. This speaks volumes. It is conclusive that diet is the principal factor in the production of the disease. Statistics, however, are unnecessary on the subject, as I feel sure the experience of every one present will corroborate the statement.

The subject of diet for children should be considered from two standpoints. First the quantity and second the quality. One is almost as important as the other. You may have as perfect food as is possible to obtain, and overfeed, and the result will be the same, as neither will be digested.

Improper food consists of two general classes, first, that which is fresh and pure, but for physiological reasons cannot be digested before dentition is completed, and other parts of the digestive system are fully developed. Second that, which when fresh and pure, is easily digested (Such as pure milk in a fresh state) but after fermentation has commenced and it has been impregnated with Pathogenic Bacteri it is unfit for use and hence improper food.

When from any cause it becomes necessary to artificially feed a child, cow's milk is most usually selected, and too often it is an impure article, fermentation having already commenced before it reaches the stomach, and often in this state it is charged with pathogenic bacterin, and if the proper temperature exists, they commence their deadly work at once. Fermented milk is one of the most prolific causes of this disease, first, because it is most frequently substituted for the breast, and second, it is the best medium for the development of bacteria found in this disease. Although fermented milk is the most frequent cause of this disease, there are various other articles of diet that produce it, but the limits of this paper forbid anything more than the mere mentioning of the fact.

TEMPERATURE.

Siebert of New York and others have made observations on the influence of atmospheric changes, upon the prevalence and mortality of this disease. The conclusion reached is, that while the disease does not depend directly or entirely on the temperature of the atmosphere, a certain elevation is, however, necessary. While the mean temperature is below 58 degrees F. the number of cases are no greater than in winter months when the temperature is low. As soon as the minimum temperature reaches above 60 degrees F. for several days in succession the disease becomes epidemic. A temperature of above 100 degrees F. is essential for the development of bacteria, as found in this disease. Bacteria may enter the gastro-intestinal tract, at a temperature below this, with perfect impunity, if the body temperature remains normal. If, however, when they enter the intestinal canal and the body temperature is elevated to 100 degrees F. or above this, they will develop and produce the same results as when the atmospheric temperature is high.

BACTERIA.

Quite a number of Pathogenic Bacteria have been found in gastro-intestinal catarrhs, but so far we are not taught any specific kind producing any distinct variety of this disease; with possibly one exception—that of the Bacillus of "Green-stool" Diarrhoea. This variety is claimed to be very contagious, as epidemics have been observed to date from the introduction of a single case into hospitals, independent of the season of the year, and attacked breast fed and artificially fed children alike. Its habitat in the small intestines, while the bacteria of other varieties of this disease inhabit the large intestines. This green color was formerly supposed to be bile coloring matter, and was thought to be due to an excess of acid in the intestinal tract; late investigations have proven both these to be false, as they do not contain any bile-coloring matter, and the reaction is alkaline, there is a defect of lactic acid. The different varieties of bacteria found in diarrhoeas of children are supposed to be due partly to the different articles of diet, and to the different stages of the disease, while others claim that all the different varieties are required to carry on their deadly work irrespective of the diet used. The truth is, our knowledge of them is so imperfect that we are not able to say just what role they do play in this regard. They are known to exist, and when the proper soil and temperature is present, they multiply and produce a chemical poison, known as Ptomains, which is a local irritant, and is readily absorbed into the circulation of the blood, which produces a true blood poison, and hence the constitutional symptoms.

These three factors, I think are essential to this disease, there are many other minor ones, such as surroundings, poverty, foreign bodies, exposure to cold, sudden change of air or water; these act principally as exciting causes, and time and space forbid anything more than mere mention of them. There is one other predisposing cause I will call attention to, one that I have not seen laid down by any writer on the subject, but from my observation is a frequent predisposing cause; I have reference to Phymosis. I have no statistics to offer you to prove this statement, as I have kept no record of the cases that I have treated of this disease; but I have some statistics which tend to verify this. According to the report of the State board of health of Alabama for the year 1888, the death rate of males from diarrhoeal diseases of children under three years old, was six per cent greater than that of the females, while the birth rate for the same year shows about two per cent more males than females. This would argue that there are more predisposing causes in the male than in the female.

The nervous reflex action of Phymosis is a well established fact, and also the influence of the nervous system over that of the digestive system, is well established and recognized. This being the case, why not recognize Phymosis as a predisposing cause of infantile diarrhoea. My attention was first directed to this some two years ago, when I was treating an infant some three or four months old, suffering with "green stool" diarrhoea. It was also developing hydrocephalus. The mother of the child was a perfect picture of health, but from some cause was not able to nurse her children. She stated when her babe was born it weighed from ten to eleven pounds, and to all appearances was perfectly healthy. When it was placed under my care at three months old, it weighed about six pounds, it was little more than skin and bones. I treated it for some two weeks, both for the diarrhoea and hydrocephalus without any improvement, in fact my little patient continued to grow worse day by day, until it had almost reached the brink of the grave; when on one occasion I was inspecting the stool on a diaper that was being removed from the little skeleton, I chanced to notice the condition of the prepuce. On close inspection I found a pin-hole opening, with firm adhesions. I at once recognized a cause of the hydrocephalus, I explained to the mother the effect it had over the nervous centres producing the water on the brain. After explaining to her the simplicity, and little danger attending the operation, she readily consented to have it circumcised. I feared that it was a hopeless case, but was satisfied that this was the only hope of giving it relief from the hydrocephalus, and decided to give my little patient the benefit of it, thinking that if I could arrest the hydrocephalus I could by other means probably, control the diarrhoea; having no thought that the circumcision would have any tendency to cure the diarrhoea. After the operation was performed, the little fellow began to improve almost from the day of circumcision, and in two weeks from date of operation he had gained six pounds, without any material change in the treatment or diet.

I have seen numbers of cases since, of a similar nature, that have ran along for months under proper dieting and treatment without improving, and as soon as circumcision was done, improvement dating from circumcision, in almost every instance. I have treated and circumcised them resulting in recovery, that I am convinced would not have recovered without the operation. I have never had but a single case that was operated upon that did not recover, and that one was delayed until nothing could have benefited it. To recapitulate briefly of the three principal factors producing this disease. First, an improper diet; this may be due to an excess of a suitable diet in a pure state, but the digestive organs are overtaxed, and as a result a fermentation is produced, which furnishes a suitable soil for the development of Pathogenic Bacterin, that find their way into the intestinal tract. But most frequently fermentation has already commenced in the food before injestion. This fermentation is dependent upon a certain elevation of temperature, which is essential to the development and growth of Pathogenic Bacteria. That when Pathogenic Bacteria enter the intestinal tract, and meet with suitable soil, they produce Ptomains, which are readily absorbed and produce local as well as constitutional disturbances. This fermentation may be acid or alkaline, and to be able to determine at the bedside which of these reactions exist, would be quite an important advance in Pediatrics, and would be of inestimable value in the treatment of the disease. It is very likely that in some cases both kinds of fermentation are going on at the same time. The reactions of the stools give no clue on this line. From the present status of investigation on this subject, our brightest prospects for light on this line is the analysis of the urine. It has been shown by different observers that Phenol, Scatol, Indol, etc., are found and excreted in the urine. However, to test urine for these products is a somewhat complicated process, but at present it is the most reliable means at our command, to determine the existence of albuminious fermentation; owing to the complication of the process of determining this fact, it is not likely to fall into general use, however we hope some more simple method may be discovered.

TREATMENT.

As diet is the most important factor in producing this disease, the first thing is to look after the food the little patient is taking; as a prophylactic there is nothing so important as food and feeding, and to produce this breast-milk from a healthy nurse is the only reliable source, all other foods are substitutes. Space forbids mentioning the various substitutes, suffice it to say that pure cow's milk from a perfectly healthy cow, fed on proper wholesome food, furnishes the best substitute; this should be used when fresh or put in bottles rendered aseptic, when first milked from the cow, and well corked and kept in a cool place; managed in this way with proper precaution it will keep perfectly fresh for several days. After the disease is well developed I doubt the propriety of giving milk in any form, even breast milk. This is especially true when the atmospheric temperature or the body temperature is high. When milk is taken into the stomach of child with high fever, it is at once coagulated into a hard indigestible mass, and is either rejected or passes down into the intestines, and adds fuel to the fire that is already going on consuming the lifeblood of the patient.

During the early stage of the disease, food of all kind should be withheld until vomiting has ceased and the gastro-intestinal tract has been thoroughly cleansed of all fermented material, then farinaceous decoctions should be given. I have found the following formula to act well in this stage: oatmeal, cracked wheat and cracked barley, in the proportion of two tablespoonsful of the former to one of the latter in a quart of water boiled down to a pint, and filtered and seasoned to taste. Of this two to three tablespoonsful are to be administered every hour or two, according to age and condition of patient. After convalescence has commenced, milk, either fresh or sterilized, or better the breast should be given cautiously, observing its effect. In the majority of cases I have found the above regimen to act well; however we are to judge each case as to the most suitable diet, as no definite rule can be laid down for each medical treatment. In the incipiency of the disease an active cathartic should be given to cleanse out the gastro-intestinal tract, of all undigested or fermented food that may be present. For this purpose I know nothing better than calomel, rhubarb and bi-carbonate soda in cathartic dose, to suit the age of the patient. This acts upon the whole of the gastro-intestinal tract, not only as a cathartic, but upon the secretions as well.

After the bowels have been thoroughly cleansed of all undigested and fermented matter and the disease does not yield, which in the majority of cases it will, if seen in the first stage, then the treatment should be antiseptic and anti-fermentive, for this the bichloride of mercury with carbolic acid, listerine and salicylate of bismuth in dose to suit age of patient, stands at the head of the list. Also the bowels should be thoroughly irrigated every eight or ten hours with antiseptic solutions. In subacute or chronic cases, this is an important part of the treatment as the lower bowels is the habitat of the bacteria found in this disease in the ordinary form; in this we only flush out the lower bowels to rid them of any offending matter, but we bring our antiseptics in direct contact with the parts affected. Irrigation of the stomach is indicated in only a limited number of cases and is rather hard to carry out in private practice. Opium and the mineral and vegetable astringents, which were once so universally used, are unscientific and irrational, and should never be given. They lock up the poison within the system and obstruct the secretions, and only hasten the fatal end. There may be a limited number of cases where a small amount of opium is admissible, where there is severe pain or extreme nervousness, but in most cases bromides and chloral control these symptoms as well as, or better, without interfering with the secretions. When opium is used at all Dovers powders is the most suitable, as it interferes less with the secretions than any other form. Stimulants should be used freely as soon as there is evidence of failure of any of the vital forces.

### TREATMENT OF "GREENSTOOL" DIARRHOEA.

As the color of the stools in this form of diarrhoea is due to a defect of Lactic acid, and is very suggestive as to the proper treatment. Lactic acid in doses of half to one grain after each feeding, I have always found to control this form of diarrhoea, without any difficulty. Last, but by no means least, if your patient is a boy, look to the prepuce; if phymosis exists, circumcise him at once and save the life of your patient.

